# Analyzing genderless fashion trends of consumers’ perceptions on social media: using unstructured big data analysis through Latent Dirichlet Allocation-based topic modeling

**DOI:** 10.1186/s40691-021-00281-6

**Published:** 2022-03-05

**Authors:** Hyojung Kim, Inho Cho, Minjung Park

**Affiliations:** Department of Fashion Industry, Ewha Womans University, 52, Ewhayeodae-gil, Seodaemun-gu, Seoul, South Korea

**Keywords:** Genderless fashion trend, Latent Dirichlet Allocation-based topic modeling, Text-mining, Social network analysis, Fashion big data analysis

## Abstract

After the development of Web 2.0 and social networks, analyzing consumers’ responses and opinions in real-time became profoundly important to gain business insights. This study aims to identify consumers’ preferences and perceptions of genderless fashion trends by text-mining, Latent Dirichlet Allocation-based topic modeling, and time-series linear regression analysis. Unstructured text data from consumer-posted sources, such as blogs and online communities, were collected from January 1, 2018 to December 31, 2020. We examined 9722 posts that included the keyword “genderless fashion” with Python 3.7 software. Results showed that consumers were interested in fragrances, fashion, and beauty brands and products. In particular, 18 topics were extracted: 13 were classified as fashion categories and 5 were derived from beauty and fragrance sectors. Examining the genderless fashion trend development among consumers from 2018 to 2020, “perfume and scent” was revealed as the hot topic, whereas “bags,” “all-in-one skin care,” and “set-up suit” were cold topics, declining in popularity among consumers. The findings contribute to contemporary fashion trends and provide in-depth knowledge about consumers’ perceptions using big data analysis methods and offer insights into product development strategies.

## Introduction

Consumers’ blogs and social network opinions have become a valuable resource for gaining marketing insights and relationship management (Zhang et al., 2009). As social media has profoundly changed our lives, the widespread adoption of social media sources has generated a vast amount of textual data. Knowledge acquired from social networks interacts with consumers and affects many companies to find their competitive advantage in improving brand products or services (Governatori & Iannella, 2011; He et al., 2013). Consumer-driven fashion trends and continuous social media monitoring has created new paradigms of trend emergence, which lead to the discovery of key values for brands. For example, the traditional runway collections’ design aspects indicated the upcoming fashion trends; however, a social media platform with real-time content posted by consumers, influencers, and brands became streamlined fashion trends (Yotka, 2020).

Trend analysis is a technique that attempts to collect information and discover patterns and estimate future predictions (Immerwahr, 2004). The fashion industry adapts the trend analysis using the text-mining technique to predict consumer nature, which is associated with business success. The growth of Web 2.0 and social networks has increased the demand for unstructured data such as news, images, and videos online. According to IBM’s report, unstructured data accounted for 93% of the total data in 2020, and it is estimated that 1.7 MB of data are generated every second (Trice, 2015). Liu et al. (2011) found that 80% of an organization’s information consists of text documents, and that using automated computer techniques is essential to exploit the knowledge from the vast amount of text. However, investing consumers’ preferences and adaptation behaviors toward fashion trends is difficult because social media text-based communication analysis is costly and complicated in processing natural language.

The fashion trend implies various societal types and numerous clothing style choices according to different types of societies. Liberal society members tend to be more accepting of radical changes and innovation, while the conservative society community prefers to maintain its conventional costume (Kawamura, 2018). South Korea is famous for its highly fashion-conscious consumers who rapidly adjust to emerging trends (Hounslea, 2019), as they are willing to engage in digital technology development (Chakravorti et al., 2020). At 87%, South Korea’s social media rate is the third highest in the world, enabling consumers to easily follow current widespread trends and to generate new information (Shim, 2020). Given that gender fluidity in fashion has seen a recent boom globally since 2018 (Menkes, 2018), the genderless concept began to expand as the trend of emphasizing gender diversity expanded in South Korea. Szmydke (2015) explained that the traditional fashion industry has been providing design and service based on gender identity; however, masculinity and femininity have diversified with the advent of genderless fashion trends. In addition to the importance of the individual’s unique taste on style, current consumers independently define and express their gender identity (Kopf, 2019). Clothing is not only a simple method to express one’s lifestyle, but also a strong tool to represent one’s characteristics. Fifty-six percent of Gen-Z consumers who have a spending power of over 140 billion dollars shop outside of their designated gendered area (Marci, 2020), and searches for the term “genderless fashion” increased by 52% (Lyst, 2019). Moreover, 51% of gender-neutral global fragrance items were launched in 2018 (Murtell, 2019), and many fashion brands promoted a campaign of diversity and inclusivity in terms of gender, ethnicity, and body image. The men’s cosmetic market has grown 1.4 times in 5 years—reaching 1.4 trillion KRW (Lee, 2019) in South Korea in response to preferences for genderless items.

Therefore, the demand for adapting the genderless fashion trend has risen among general consumers and gender-neutral apparel has strode into retail prominence. Although very few studies have analyzed fashion trends in consumer behavior using the text-mining technique (Blasi et al., 2020; Rickman & Cosenza, 2007), no previous studies have focused solely on consumers’ perceptions of genderless fashion trends. Moreover, many researchers have explored genderless fashion in terms of design style elements, collection image characteristics, and sociocultural impact (Jordan, 2017; Rocha et al., 2005; Shin & Koh, 2020; Xu & Li, 2012) through qualitative research methods. The prominent genderless fashion trend is increasing, and the massive amount of big data has made it possible to understand consumers’ requirements and demands. To the best of our knowledge, this is the first study to evaluate consumers’ awareness of the current genderless fashion trend using the text-mining method. Therefore, this study demonstrates the genderless fashion trend perception among consumers on social media by applying textual data. More specifically, this research aims to answer the following questions.

1. What major keywords do consumers use when commenting on genderless fashion?
2. What are the main topics of genderless fashion and how do consumers perceive it?
3. How have the genderless fashion trends changed over time?

To investigate the research questions, we utilized a probabilistic topic modeling approach known as Latent Dirichlet Allocation (LDA; Blei et al., 2003; Griffiths & Steyvers, 2004; Newman & Blocks, 2006) for consumers’ narrative postings on community sites such as blogs and online communities. LDA-based topic modeling is a supervised machine learning algorithm used to extract latent topics from the thematic structure of large volumes of texts (Elgesem et al., 2015). The computational content-analysis of LDA-based topics enables the classification of large amounts of unstructured text documents. Consequently, LDA-based topic models are efficient in discovering and describing hidden semantic structures in a collection of texts (Koltsova & Shcherbak, 2015). In particular, we analyzed the search keyword “genderless fashion” on the portal site NAVER (http://www.naver.com)—the largest web search engine in South Korea. We then examined consumers’ perceptions of genderless fashion over the past 3 years by collecting their texts from blogs and online communities. After the data cleansing and preprocessing procedures, we specified the top keywords to extract the topics. Then, an n-gram analysis (Wallach, 2006) was applied to categorize the continuous sequence of high-order phrases from the morphologically analyzed texts. To define the number of topics, perplexity and coherence tests were examined for interpretability verification. The intertopic distance map (IDM; Blei et al., 2003) was used to determine the similarity of the chosen topics using a graphic plot showing the specific gravity of the topic and the distance among the topics. Finally, the selected topics were labeled and compared with the representative documents, and a time-series analysis was performed to measure the topic trend change.

This study advances our in-depth understanding of genderless fashion trends and contains diverse perspectives on consumers’ behaviors and interests. This study explains how fashion trends are perceived and commercialized, related to consumers’ use of social media. Further, we extend our research on fashion trend analysis by applying text-mining algorithms to extract the most relevant topics, which goes beyond the findings in the existing literature. Despite the high level of demand among consumers in the pursuit of acceptance of various gender identities in the fashion industry, relevant studies are scarce. In this context, research on genderless fashion trend analysis based on a consumer-driven text-mining analysis is essential, and the current findings will enable fashion brands to forecast customers’ preferences for purchasing gender-neutral products and develop marketing strategies through social media channels.

## Literature review

### Genderless fashion trend

The genderless fashion phenomenon has recently emerged as a new standard and has been cited as a major trend among consumers (Bernard, 2018; Kerpen, 2019; Segalov, 2020). The term “genderless” is also referred to as “agender,” “gender fluidity,” “gender neutral,” “gender diversity,” and “gender-free”—all of which refer to the state of being without a clear gender identity (Robinson, 2019). It refers to using products and creating styles according to individual personality and taste from a neutral perspective, regardless of gender. Most societies define traits specific to a gender and orient their members in that direction (Risman & Davis, 2013); however, genderless is interpreted as a movement to remove the social division between women and men and regard them as neutral individuals. For example, the binarity of gender was classified into distinct male and female segmentations, producing various stereotypes and corresponding behaviors. Strict adherence to traits of masculinity and femininity were expected from each sex, and costumes reflected the resulting dichotomous social norms. The perception of gender was influenced by factors such as feminism and relevant social movements in the 1960s and the development of mass media and the change from biological sortation to social gender. This had an impact on “androgynous” styles in the 1970s and “glam” looks in the 1980s, which transformed into the “unisex” concept, described as suitable for both males and females (Bardey et al., 2020; Mills, 2015). Lee (2021) highlighted that unisex is different from genderless fashion in terms of distinguishing methods to differentiate gender; it is based on the gender distinction between men and women, and embraces the same design, whereas the genderless style does not dichotomize gender and encompasses a wide spectrum of gender identities.

Millennials and Generation Z have different values and lifestyles than the previous generations, particularly in relation to the traditional gender role distinction. As the leading groups of trends and consumption, they want to define and express their gender identity on their own because of their great desire to express their social influence and external images (Wertz, 2018). A recent survey indicated that 38% of Generation Z and 27% of Millennials, who will account for $143 billion purchasing power in the next 4 years (Anyanwu, 2020), agreed that an individual cannot be judged or determined by gender. With this in mind, high-end brands projected runway models indistinguishable in terms of gender, while masstige brands introduced retail strategies to eliminate the distinction between men’s and women’s products in stores or launched new public brands. In addition to women’s and men’s wear brands, one major children’s wear brand removed boys’ and girls’ labels from the store floor plan to reinforce the extensive product choice preferences (Newbold, 2017), eliminating gender stereotypes for their customers.

### Text-mining analysis

Text-mining is an artificial intelligence technology that utilizes natural language processing to obtain meaningful information from vast unstructured textual data (Liu et al., 2011; Nishanth et al., 2012) or to estimate uncertain patterns (He et al., 2013). It includes the processes of editing and organizing several documents composed of words, characters, and terms (Nishanth et al., 2012). As a big data analytics extension technique, text-mining analysis examines large and varied data documents to uncover nontrivial information such as unknown correlations, customer preferences, and market trends that aid in the best decision making in the business (Hashimi et al., 2015). In particular, after the rapid increase in social network services, social media mining has been adopted to understand and interact with customers and gain a competitive advantage. According to Reports and Data (2020), the text-mining market will reach $16.85 billion by 2027 owing to the high rise in the adoption of social media platforms, and many business organizations have deployed text-mining analytics to transform data into competitive knowledge.

Many previous researchers have used text-mining techniques to analyze consumers’ brand sentiments (Mostafa, 2013), to measure consumer preferences (Rahman et al., 2014), and to survey the commerce trend on social media (Shen et al., 2019). Regarding fashion, Lang et al. (2020) evaluated consumers’ fashion-renting experiences through in-depth text analysis using LDA-based topic modeling, and Dang et al. (2016) classified fashion content texts from social networks using a support vector machine. Choi and Lee (2020) researched ethical fashion using text-mining with network analysis, and Lee et al. (2018) analyzed luxury fashion brands and mass brands’ evaluations of Twitter messages. Owing to the strong capabilities of text-mining techniques, many attempts have been made to analyze social media content to yield valuable findings on consumers’ behavior and sentimental values toward a brand. However, previous studies have dealt with relatively limited information, focusing solely on consumers’ perceptions of genderless fashion trends. Consequently, to analyze mainstream fashion trends and understand consumers’ interests, a text-mining method was employed for this study.

### LDA-based topic modeling

**Fig. 1:**
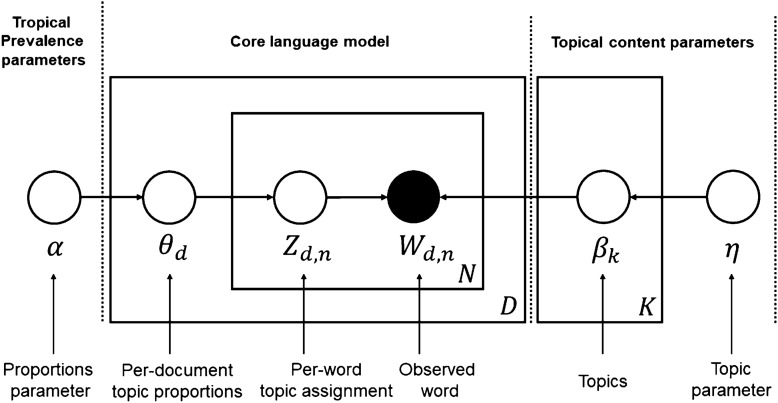
Graphics of document generation for LDA algorithm (Blei, 2012, p. 81)

In this study, LDA-based topic modeling (Blei et al., 2003) was utilized to extract customers’ perceptions of the genderless fashion trend on social media. Topic modeling allows the user to detect and summarize latent semantic structures, and LDA is the most common method for clustering abstract topics that occur in a collection of documents (Nabli et al., 2018). LDA assumes that documents consist of a mixture of topics, and that topics generate words based on probability distributions. As shown in Fig. 1, Blei (2012) explained the LDA model algorithm as follows: the square boxes are called “plates” and “N” stands for a collection of words collected within a document, “D” for a collection of documents, and “K” for a set of topics. The circles represent probability parameters, and the node “${W}_{d,n}$” is observed as a word in the document; while topics, topic distributions, and topic assignments are not revealed. There are full words (“${W}_{d,n}$”) in the numerous documents (“D”) collected by the researchers, assuming that each word has a corresponding topic (“${Z}_{d,n}$”).

There are many different topics embedded in each document, and the distribution of topics differs. Therefore, LDA deduces the latent variables of the document through the words contained in the document and generates a specified number of topics from the document stack through the Dirichlet distribution. In this study, LDA-based topic modeling was adopted to understand the consumer-driven content of genderless trends in social media networks. Various researchers have explored LDA-based topic modeling to discover new knowledge about consumers’ communication. Bastani et al. (2019) analyzed the customer complaints of national financial agencies, and fashion design participants were analyzed to observe research trends (Jang & Kim, 2017). Gray et al. (2015) developed an LDA-based text-mining methodology to define fashion styles obtained from online apparel information with affiliate networks. In contrast to the approach of consumers’ research in the fashion industry conducted in the various studies discussed above, genderless fashion trend research is unknown. Therefore, we developed a primary approach to discover consumers’ preferences and interest in social media toward the genderless fashion trend with an LDA-based topic modeling proposal.

## Methods

### Data collection

We obtained data from the largest Korean search portal engine—NAVER—focusing on consumers’ online community and blog reviews for 3 years since the genderless fashion trend began (Menkes, 2018): from January 1, 2018 to December 31, 2020. To gain insights related to genderless fashion trends among consumers’ posts and communication, a search of the keyword “genderless fashion” was conducted, which produced 9722 posts. The web crawling program language Python 3.7 (http://www.python.org) was used to build the model. Consumers’ posting date, platform type, title, contents, and link information were gathered; the text-mining objects were title and content. Data were pre-processed to cleanse them of undesirable words, special characters, non-Korean words, and punctuation. Afterward, word tokenization was lemmatized and converted into the minimal unit of meaning formats such as nouns, adjectives, verbs, or adverbs in their dictionary forms. These words were accumulated in the bag-of-words model (Zhang et al., 2010), which represents a multiset of words regardless of word order. Words that occurred in 80% of the documents and in fewer than five documents were removed (Jauhari et al., 2020). Moreover, search keywords’ implied synonym words such as gender-neutral, gender fluid, gender diversity, and fashion that could have affected the results, were removed. Hence, only meaningful words relevant to the generation of the topics remained.

### Measurement and research process

**Fig. 2:**
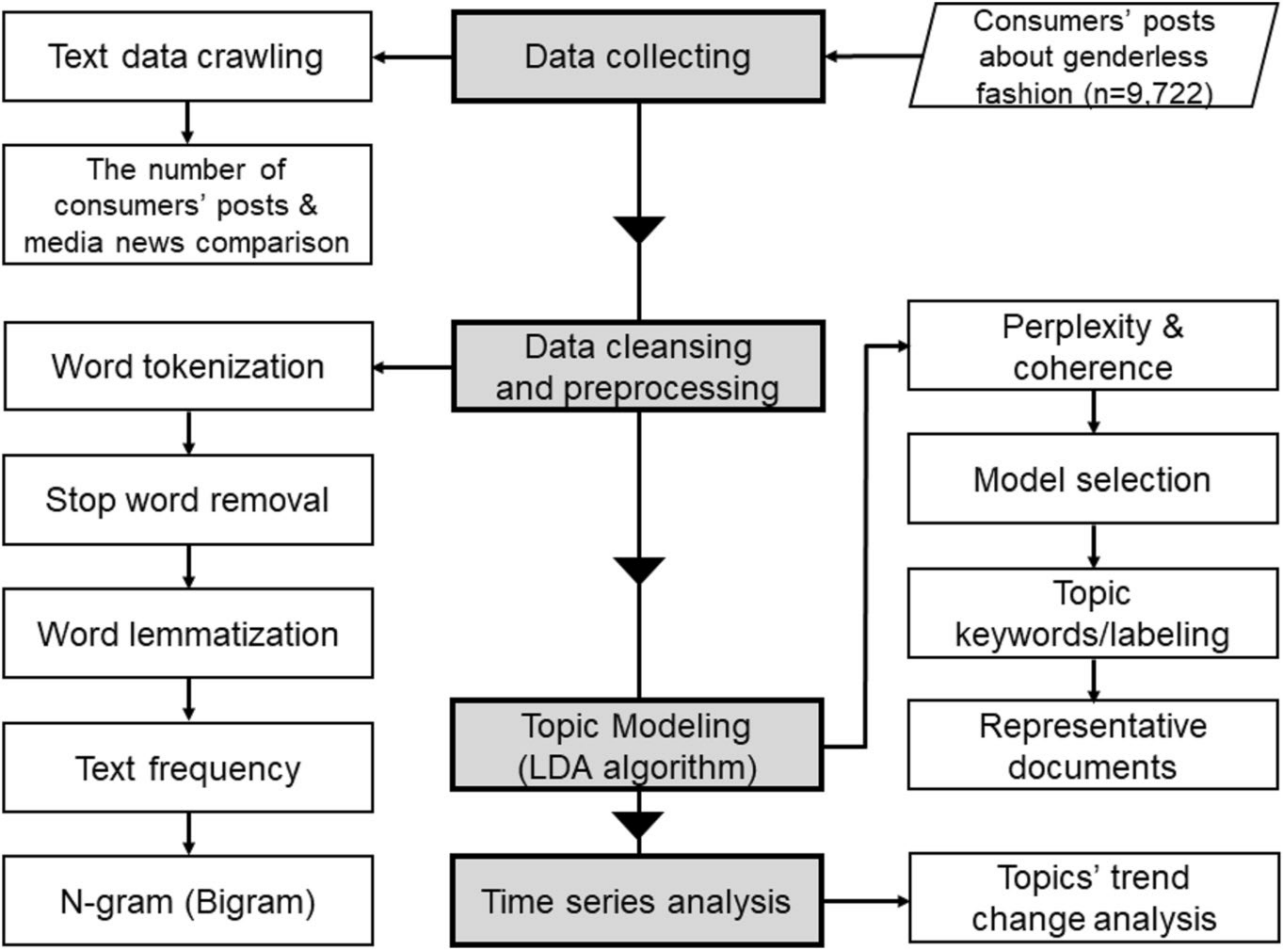
Research data processing flowchart

To perform the research data analysis, we used Python 3.7 to perform data processing and applied LDA-based topic modeling. The detailed research process flowchart, performed over four steps, is shown in Fig. 2. First, the web crawling technique was performed using the keywords “genderless fashion” to collect consumers’ review posts on NAVER’s blogs and online communities. The number of keyword changes over 3 years was evaluated to estimate consumers’ preferences and interests. Second, data cleansing and preprocessing of unstructured text data were conducted to eliminate irrelevant or generic words. Consequently, the entire text document was into split into individual words, which is known as word tokenization. Then, stop-word removal and word lemmatization were applied to filter meaningful words on natural language data. For example, onomatopoeic words (“haha,” “nope,” etc.), emoticons, propositions (“the,” “a,” etc.), inappropriate words (“recently,” “more,” “really,” etc.) were removed. Then, the top 50 text frequency words and bigram rates were analyzed. Next, to analyze topic modeling based on the LDA algorithm, a topic model number was defined by applying the measure of perplexity and coherence parameters. Then, each topic model’s labeling was selected based on the observed keywords and representative documents associated with the high weight of the topic. In this step, an IDM was applied to determine the degree to which each topic was related to other topics and the degree of similarity between topics. Fourth, to measure the topic trend change over the past 3 years, we investigated the number of consumers’ posts containing each topic. Subsequently, a time-series linear regression analysis was performed to confirm the annual trends of the topic.

## Results

### Status of consumers’ posts and media news about genderless fashion

**Fig. 3:**
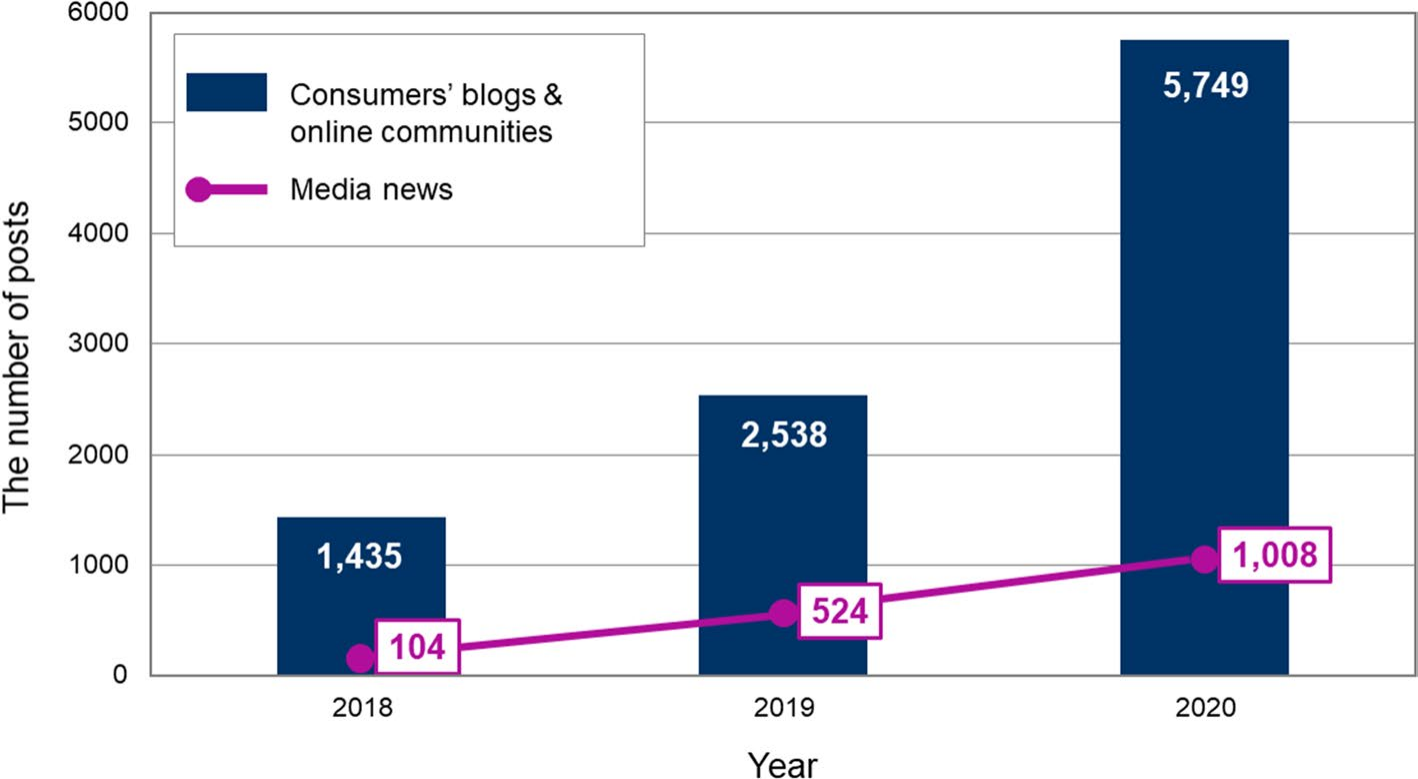
The number of consumers’ posts and media news for 3 years (2018–2020)

We compared the number of posts over 3 years from 2018—when genderless fashion was cited as a major trend—to 2020 by crawling consumers’ blog and online community posts on NAVER and media news posts. In these 3 years, 9722 pieces of consumer-generated content about genderless fashion were uploaded, and the yearly trend showed that the number of online posts had steadily increased: 1435 postings in 2018, 2538 postings in 2019, and 5749 postings in 2020. Consistently, there were 104 online news articles in 2018, 524 in 2019, and 1008 in 2020. As shown in Fig. 3, both consumers’ and media news outlets’ posts continued to increase, especially in 2020, when it doubled compared to 2019. Therefore, it was confirmed that consumers’ interest in genderless fashion has grown rapidly.

### Text frequency 

**Fig. 4:**
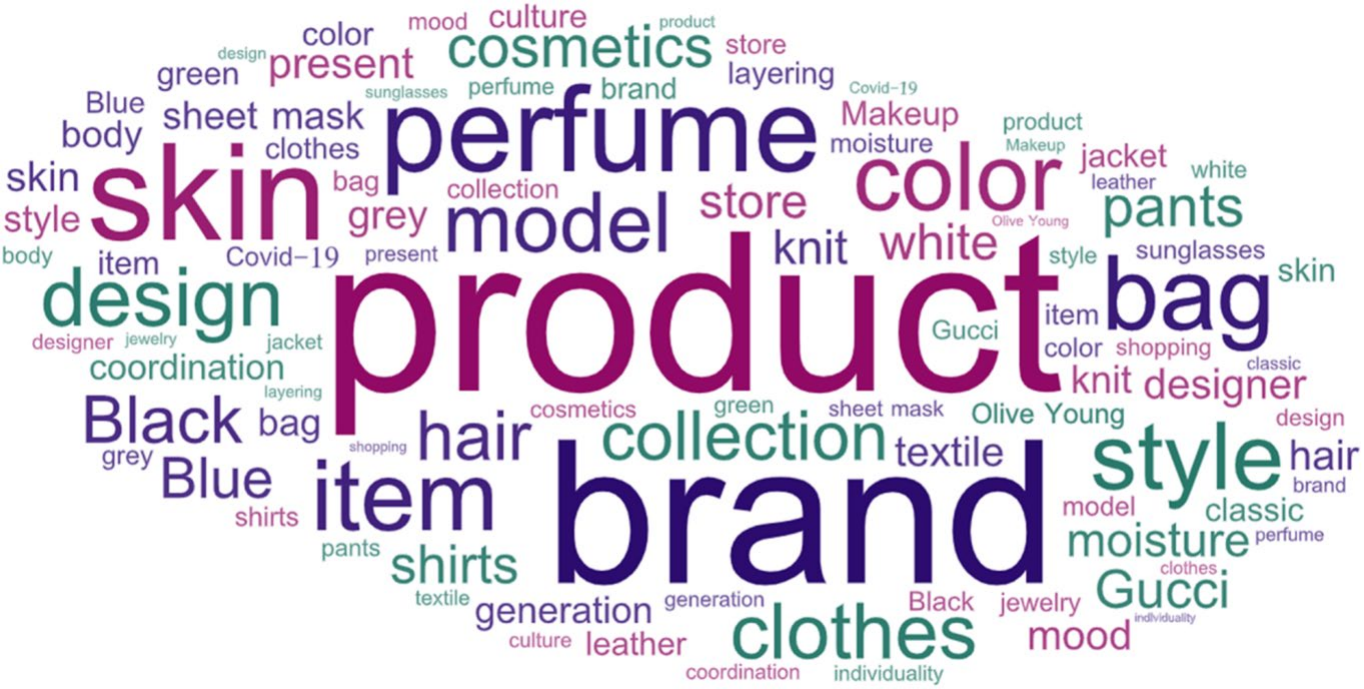
Word cloud visualization results

To analyze the key terms related to genderless fashion, we combined the titles and contents of consumers’ posts. Data cleansing and preprocessing were essential for generating meaningful topic modeling. We performed word tokenization to analyze the text dataset as a morpheme, turning it into the smallest unit of meaning through natural language processing (Bastani et al., 2019). To filter out unnecessary words, stop-word removal (Nabli et al., 2018) was conducted, eliminating undesirable fragments such as punctuation, single-letter words, grammatical errors, and numbers. The resulting set was extracted with only nouns and adjectives after word lemmatization, maintaining the basic dictionary form of a word after removing the inflectional endings. Accordingly, the frequency value of the occurrence of all extracted words was obtained, except for the words that appeared more than 80% of the time or in less than five documents. The top 45 keywords based on the extracted frequency are listed in Table 1. The results of visualizing the top 50 of the highest frequency keywords from 4051 word lists is shown in Fig. 4. Words with a high frequency of occurrence are expressed as bigger and bolder in the word cloud. To review the top-ranking frequency occurrence words, genderless fashion-related brands (e.g., Gucci, Olive Young) and merchandise (e.g., product, design, style, item, bag, pants, shirts, store, jacket, sunglasses, knit) were extracted in the fashion and beauty industry (e.g., clothes, cosmetics, hair, makeup, jewelry). Concerning color, black was the highest, followed by white, blue, gray, and green (in order).

**Table 1:** Results of the word frequency analysis (n = frequency)

Rank	Word	*f*	Rank	Text	*f*	Rank	Text	*f*
1	Product	20,871	16	Black	4596	31	Jacket	2374
2	Brand	15,731	17	Moisture	4258	32	Sunglasses	2267
3	Perfume	13,160	18	Designer	3574	33	Layering	2186
4	Skin	13,120	19	Generation	3330	34	Leather	2089
5	Color	11,397	20	Pants	3327	35	Body	2060
6	Design	10,521	21	Present	3254	36	Culture	1975
7	Style	8960	22	Hair	3140	37	COVID-19	1771
8	Clothes	6400	23	Gucci	3126	38	Textile	1703
9	Bag	5848	24	Shirts	2755	39	Classic	1622
10	Collection	5822	25	White	2552	40	Shopping	1608
11	Item	5623	26	Olive Young	2508	41	Grey	1605
12	Model	5452	27	Blue	2496	42	Knit	1596
13	Cosmetics	5011	28	Store	2450	43	Green	1557
14	Sheet mask	4929	29	Makeup	2432	44	Jewelry	1518
15	Coordination	4784	30	Mood	2384	45	Individuality	1386

### N-gram analysis 

**Fig. 5:**
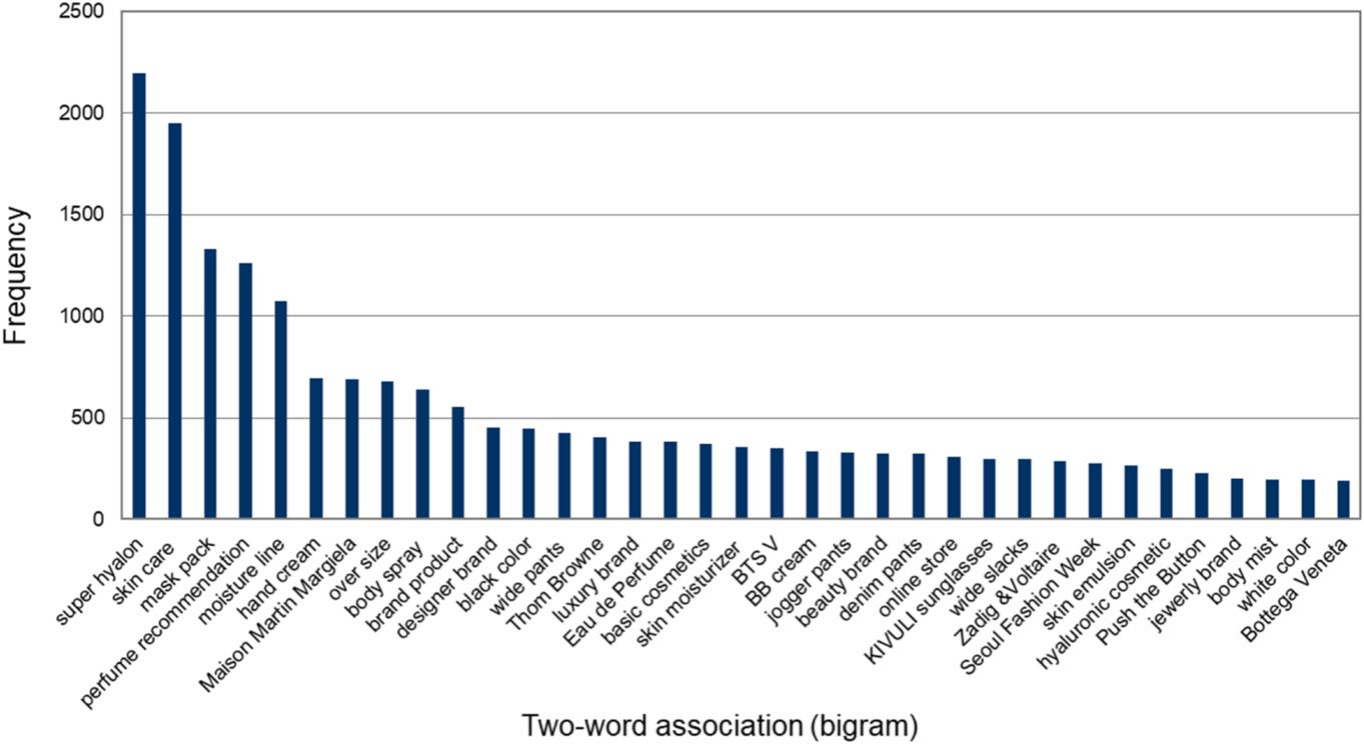
Results of the bigram analysis

We attempted to improve text classification by determining which words were connected in the unigram dataset. Bigram means that two-word phrases belong to the n-gram analysis method to generate contiguous word pairs in the corpus and gain the contextual word association (Crossley & Louwerse, 2007). It is also useful to compare bigrams in two different sentences because it allows us to identify the similarities and various types of words in context. The results of the top 35 bigrams from 6703 two-word lists are shown in Fig. 5. Cosmetics (e.g., super hyalon, skincare, mask pack, moisture line, hand cream, basic cosmetics, skin moisturizer, BB cream), fragrance (e.g., perfume recommendation, body spray, Eau de perfume), fashion brands (Maison Martin Margiela, Thom Browne, Zadig & Voltaire, Push the Button, Bottega Veneta), and style-related items (oversize, wide pants, jogger pants, denim pants, wild slacks) appeared accordingly.

### Select the optimized number of topics

**Fig. 6:**
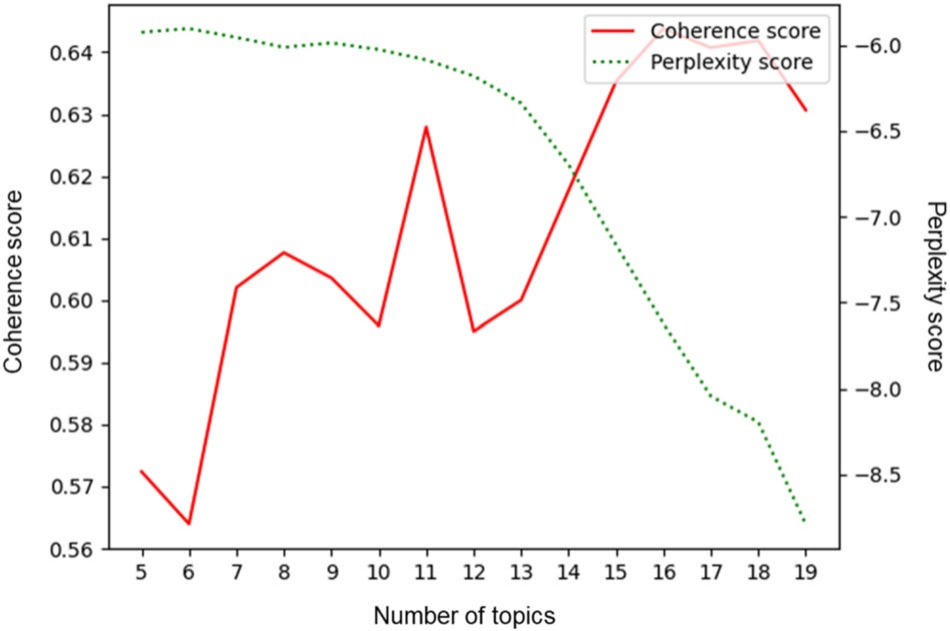
The interpretability of topic modeling

We analyzed the coherence score and perplexity score to evaluate the optimal number of topics as quantitative diagnostic metrics. The coherence score measures how frequently the top keywords of each topic co-occur to identify which of the top words contributes the most relevant information to the given topic (Blair et al., 2020). The perplexity score is an indicator of whether the topics are clearly classified, and it is assumed that the smaller the value, the better the actual literature results reflected by that topic (Inglis & Foster, 2018). Therefore, the smaller the perplexity value and the larger the coherence value, the more semantically consistent the topic model that is constructed. By calculating the perplexity and coherence values for all the words in the web-crawled document, we ensured that the LDA-based model achieved maximum coherence score and minimum perplexity score with the number of topics (*k* = 18; Fig. 6).

### Topic selection and labeling

**Fig. 7:**
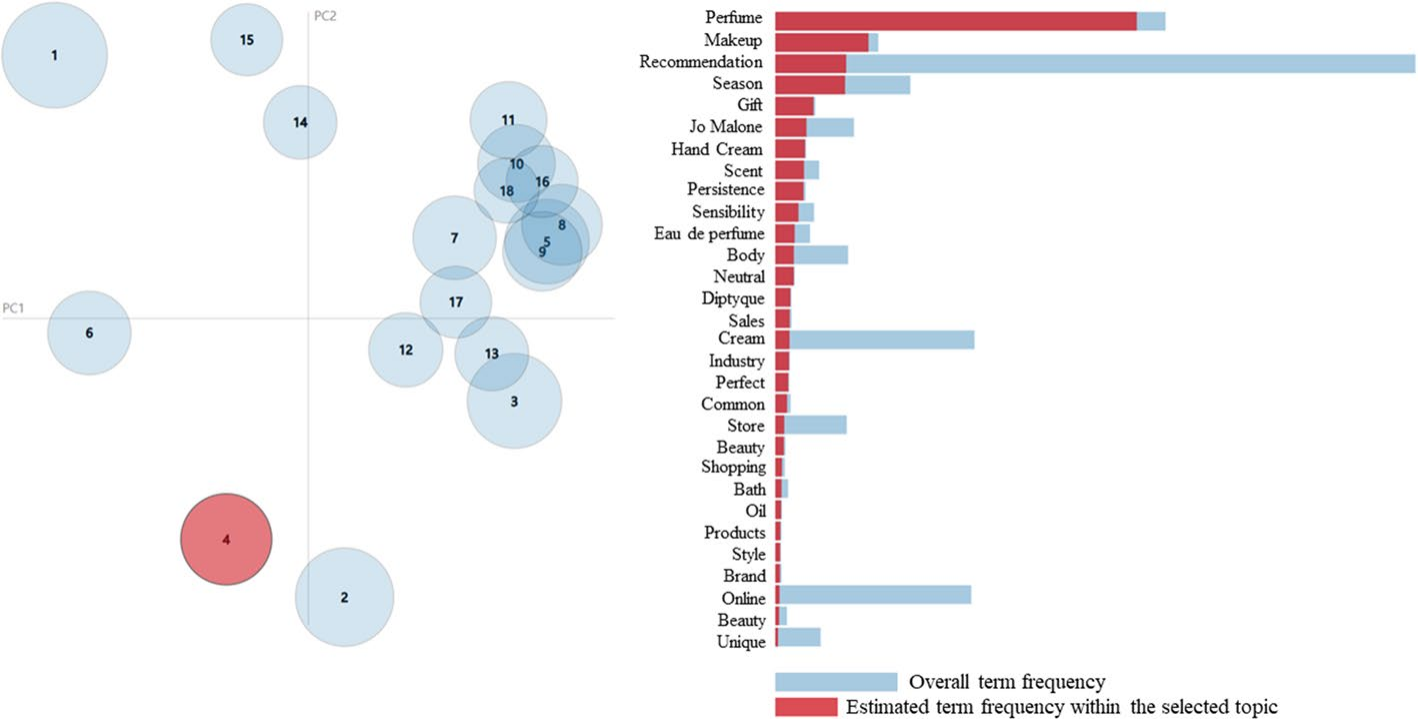
Intertopic distance map (IDM) of genderless fashion LDA topic modeling

The IDM for topics extracted from topic modeling in this study is shown in Fig. 7. IDM is a diagram that shows the weight of a topic and the distance between topics, and makes it possible to understand the degree of relevance of each topic to other topics (Sievert & Shirley, 2014). The topic view is on the left, and the term bar charts with 18 topics selected are on the right. Selections are linked so that the researcher can briefly demonstrate the aspects of the relationship of the topic terms. The distribution of topics related to the subject shows that the proportion of each topic is similar, which confirms that the deviation is non-significant. Furthermore, because the topics do not altogether overlap with each other, the association between the topics is low, which means that each topic is divided into a relatively clear research area.

We classified consumers’ perceptions of genderless fashion for 3 years by identifying keywords derived using LDA-based topic modeling algorithms and documents with a high weight of the topic. Table 2 shows the topic number corresponding to the keywords of the topic, the weight of the topic in the document, the date of the posting, and the title as an example from topic number one to five.

**Table 2:** The representative documents based on the topic weight

Topic #	Weight	Date	The title from the blog or online community posts
1	7.12	2020-06-10	VT Super Hyalon 2nd product review—No. 1 item moisture line for Musinsa.com beauty
2	9.35	2019-12-09	2020 Men’s brand-new jewelry trend—gender neutral
…	…	…	…
17	9.59	2019-06-02	I went to the Sunglasses brand KIVULI event and came back with a crush!
18	6.74	2020-08-22	Stella McCartney Launches Genderless 'Shared' Capsule Collection

The topic labeling process was discussed and confirmed by five experts in the fashion and textile industries. Table 3 represents 18 topics and top keywords for genderless fashion trend topic modeling in accordance with the analysis of major keywords and documents with high topic weight in previous works.

**Table 3:** Topic modeling analysis result about the genderless fashion

Topic #	Topic name	Top 10 keywords
1	Moisturizing skin care	Super Hyalon, LAKA, Sheet Mask, Moisture, Toner, Care, Brand, Product, Recommendation
2	Summer Jewelry	Summer, Ring, Earrings, Jewelry, Handmade, Minimalism, Chic, Trend, Item, New arrival
3	Men's fashion & grooming	Thom Browne, JW Anderson, Apparel, Shoes, Store, Street fashion, Men's cosmetics, Olive Young, Skin care, Mist
4	Perfume and scent	Jo Malone, Diptyque, Eau de perfume, Scent, Persistence, Neutral, Season, Common, Sensibility, Gift
5	Hairstyle & Color	Hairstyle, Hair salon, Short cut, Feminism, Celebrity, Color, Mint, Pastel, Romantic, Neutral
6	High-end fashion's basic item	Maison Martin Margiela, Luxury, Collection, Daily, Basic, T-shirt, Cardigan, Unisex, Shopping
7	Cosmetic beauty brands	Cosmetics, Beauty, Makeup, Gender-free, Brand, Product, Taste, Purchase, Industry, Market
8	Bags	Bag, Purchase, Tote bag, Size, Pattern, Push the Button, Bean Pole, Juun. J, Fashion Week, Collection, Point, Look book
9	FW Fashion	Autumn, Jacket, Loafers, Blue, denim, coordination, recommended, match, BTS, culture
10	Collaboration	Celine, Hedi Slimane, Nike, Collaboration, Retro, Kang Daniel, Givenchy, Look book, Popularity, Unique
11	Genderless concept models	Prada, Collection, Main model, Chic, New York, Discrimination, Prejudice, Tony Moly, Charisma, Chic
12	Luxury brand sunglasses	Gucci, Saint Laurent, Dior, Luxury goods, Sunglasses, Domestic, Department stores, Pop-up stores, Suggestion, Preference
13	Body spray	Body spray, Niche, Musk, Eco, Sensation, Review, Reaction, Zardic & Voltaire, Neutral, Gift
14	Pants style	Wide pants, Skirt pants, Suit, Goods, Boots, Comfort, Layering, Styling, Concept, Diversity
15	All-in-one skin care	All-in-one, Cosmetics, Men, Skin, Care, Lotion, Emulsion, Launch, Proposal, Directing
16	Set-up suit	Female, Set-up suit, Custom, Suit, Shirt, Popular, Trendy, Sensibility, Border, Comfort
17	Domestic eyewear brand	KIVULI, Eyewear, Sunglasses, Label, Designer, popularity, Recommendation, Retro, Market, Trend
18	Capsule collection	Capsule collection, Launch, Balenciaga, Millennial, Men, Pink, Change, Consumer, Equality, Lifestyle

### Time-series analysis

To understand the trend of each topic by year, the year was applied as the independent variable, the weighted average value of the topic by year was used as the dependent variable, and a series linear regression analysis was performed. In addition, the values of the regression coefficient and the significance probability of the linear regression analysis were verified as criteria for judging the rise and fall of trends by year. Only those topics with a significant p-value (< 0.05) and a Durbin–Watson value greater than 1.5 and less than 2.5, if the regression coefficient value was positive, were classified as a “hot topic”; while, if negative, they were classified as a “cold topic,” and topics for which no meaningful result could be derived were classified as a “neutral topic” (Griffiths & Steyvers, 2004).

The hot topic that has been rapidly growing among consumers in the genderless fashion trend for the last 3 years was “perfume and scent.” In contrast, “bags,” “all-in-one skin care,” and “set-up suit” (i.e., a casual outfit that can be used together or worn separately with a jacket and pants) were cold topics, indicating that consumers’ interest in these gradually declined. The remaining topics were classified as neutral topics because they were non-significant in the time-series analysis (see Table 4).

**Table 4:** Result of the topic trend change after time series linear regression analysis

Topic	Graph	Statistical indicators	
[Topic 1] Moisturizing skin care		Coefficient P-value Durbin-Watson Hot/Cold	2.90 .34 2.76 Neutral
[Topic 2] Summer Jewelry		Coefficient P-value Durbin-Watson Hot/Cold	− 2.54 .16 3.53 Neutral
[Topic 3] Men's fashion & grooming		Coefficient P-value Durbin-Watson Hot/Cold	1.20 .32 2.78 Neutral
[Topic 4] Perfume and scent		Coefficient P-value Durbin-Watson Hot/Cold	2.09 .04 2.41 Hot
[Topic 5] Hairstyle & Color		Coefficient P-value Durbin-Watson Hot/Cold	− .92 .25 1.69 Neutral
[Topic 6] High-end fashion's basic item		Coefficient P-value Durbin-Watson Hot/Cold	.21 .13 1.64 Neutral
[Topic 7] Cosmetic beauty brands		Coefficient P-value Durbin-Watson Hot/Cold	.28 .56 3.18 Neutral
[Topic 8] Bags		Coefficient P-value Durbin-Watson Hot/Cold	− .61 .02 2.35 Cold
[Topic 9] FW Fashion		Coefficient P-value Durbin-Watson Hot/Cold	.08 .91 1.50 Neutral
[Topic 10] Collaboration		Coefficient P-value Durbin-Watson Hot/Cold	− .17 .83 2.12 Neutral
[Topic 11] Genderless concept models		Coefficient P-value Durbin-Watson Hot/Cold	− .29 .69 2.50 Neutral
[Topic 12] Luxury brand sunglasses		Coefficient P-value Durbin-Watson Hot/Cold	− .28 .61 1.46 Neutral
[Topic 13] Body spray		Coefficient P-value Durbin-Watson Hot/Cold	.55 .66 2.86 Neutral
[Topic 14] Pants style		Coefficient P-value Durbin-Watson Hot/Cold	− .02 .96 2.60 Neutral
[Topic 15] All-in-one skin care		Coefficient P-value Durbin-Watson Hot/Cold	− 1.02 .02 2.21 Cold
[Topic 16] Set-up suit		Coefficient P-value Durbin-Watson Hot/Cold	− .21 .01 1.70 Cold
[Topic 17] Domestic eyeglasses brand		Coefficient P-value Durbin-Watson Hot/Cold	− .69 .77 3.12 Neutral
[Topic 18] Capsule collection		Coefficient P-value Durbin-Watson Hot/Cold	− 1.14 .09 2.55 Neutral

## Discussion

The concept of gender diversity has begun to expand with the trend of focusing on individuals’ unique taste importance. Consumers began to self-define and express their gender identity and discuss it through social media channels. With access to massive amounts of unstructured data from blog and online community reviews, the purpose of this study was to identify consumers’ perceptions and preferences regarding genderless fashion based on the text-mining analysis approach. In particular, we selected the LDA-based topic modeling method to examine a large amount of qualitative information obtained from consumers’ posts.

Text data were collected from the search keywords “genderless fashion” on the NAVER portal site from January 1, 2018 to December 31, 2020. A total of 9722 postings were collected, word tokenization was conducted after data preprocessing and cleansing, and word frequency and n-gram analysis were performed to remove stop words. To determine the optimal number of topics, perplexity and coherence scores were evaluated, and 18 topic keywords were finally selected through the LDA algorithm analysis. To select each topic, the contents of the representative documents with a high weight of the topic were reviewed. Finally, a time-series regression analysis was performed to understand the trend of topics by year, and the hot topic of the uptrend and the cold topics of the downtrend were selected.

First, to review the text frequency and n-gram analysis results, our study findings revealed that consumers are interested in external images as independent individuals rather than meeting other people’s standards, and often talk about fashion brands (“Gucci,” “Maison Martin Margiela,” “KIVULI,” “Zadig & Voltaire,” “Push the Button,” “Bottega Veneta”), items (“clothes,” “bag,” “pants,” “shirts,” “jacket,” “sunglasses,” “jewelry,” “style”), and cosmetic and perfume products (“skin,” “sheet mask,” “skin moisturizer,” “makeup,” “body spray,” “body mist,” “hand cream,” “Eau de perfume,” “Super hyalon,” “BB cream,” etc.) related to genderless fashion. This indicates that consumers’ recommended products and styles of genderless fashion are affected by the diverse fashion labels collection. Consumers are interested in the coordination and design details of certain brand items related to the gender-neutral concept. In particular, beauty cosmetics and fragrances, which are dominated by female-oriented stereotypes, are now being highlighted, regardless of gender division, owing to the influence of genderless fashion trends among consumers. Kim (2021) stated that many brands are launching gender-free cosmetics, which has become an opportunity for male consumers’ interest in skin care to become specialized. These results can be understood in the same context as those of previous studies (Newbold, 2017; Reis et al., 2018)—that genderless fashion is a response to the needs of the fluid market niche increase aside from femininity and masculinity stereotypes. Wertz (2018) also indicated that Millennials and Generation Z’s consumption trends value individuality and practicality rather than gender. Concerning color, An (2018) as well as Hong and Joo (2020) mainly pointed out that “pink” was the trendy color on gender-neutral menswear collections; however, we discovered that achromatic colors such as “black,” “white,” “gray” mentioned mostly among the consumers. The results suggested that the men’s collection combines colorful colors into genderless fashion, but our study confirmed that consumers prefer dark colors.

Second, 18 topics were analyzed from LDA-based algorithms and 13 topics were classified as fashion categories (i.e., “summer jewelry,” “men’s fashion & grooming,” “hairstyle & color,” “high-end fashion’s basic item,” “bags,” “FW fashion,” “collaboration,” “genderless concept models,” “luxury brand sunglasses,” “pants style,” “set-up suit,” “domestic eyewear brand,” and “capsule collection”), while 5 topics were classified as beauty and fragrance categories (i.e., “moisturizing skin care,” “perfume and scent,” “cosmetic beauty brands,” “body spray,” and “all-in-one skin care”). The fashion industry has provided designs and services differently according to gender (Szmydke, 2015); however, new product development and rebranding strategies have emerged in accordance with the gender fluidity change followed by consumer-driven change. Previous studies (Hong & Joo, 2020; Shin & Koh, 2020) have investigated genderless fashion in terms of design and style based on the collection images. Kim (2020) and Yang (2020) researched genderless trends in cosmetic brands’ advertisements. Hence, our results indicated that the main interest in the genderless concept of current consumers lies in the fashion and beauty fields by expanding existing qualitative studies using big data. In particular, South Korea’s male cosmetics consumption is number one in the global market (Im, 2016), which is consistent with our results. The effect of the gender fluidity phenomenon on the beauty industry was also revealed in our results (e.g., “super hylaon,” “LAKA”) as the genderless-only cosmetic brands.

Third, our time-series linear regression analysis revealed a hot topic (“perfume and scent”) and three cold topics (“bags,” “all-in-one skin care,” and “set-up suit”), while the rest were presented as neutral topics. The topic that has continuously grown among consumers in relation to the genderless fashion trend in the last 3 years has been “perfume and scent.” Certain brands of seasonal perfumes (“Jo Malone,” “Diptyque”) and scents (“Eau de perfume,” “common”) were mentioned among the consumers. As “gift” suggests in the topic, genderless fragrances have a sensuous and soft scent, which are easy to give as a present regardless of gender. According to BBC’s report, gender fluid fragrances have surged in popularity, increasing to 51% as compared to 17% in 2010 (Bolongaro, 2019). In contrast, “all-in-one cosmetics” attracted high consumer interest in the beginning, but they gradually declined in popularity. The high demand for “moisturizing skin care” indicates that male consumers used all-in-one products because of their convenient usage in the past; however, now they can choose exclusive genderless products, allowing them to choose their own products by function and purpose (Hong, 2020).

Considering the changes in consumers’ perception of fashion products, interest in bags has been declining. “Tote bags” and “size” were considered because of users’ light-weight concerns, and they referred to the brand look-book (“Beanpole”) or fashion week collection (“Juun. J,” “Push the Button”). Yoo (2020) explained that handbag brands have expanded the range of tote bags, particularly because of their unique characteristics as well as the effect of genderless fashion trends. The “set-up suit” topic also showed a steady decline. Business casual suits are tailored (“custom”) or users prefer practical styling with a comfortable pattern (“comfort”) along with the demand for female consumers. Demand for women’s suits increased with the growth of genderless fashion, but it seems that the demand has decreased owing to the recent increase in telecommuting under the influence of the COVID-19 pandemic.

## Conclusions

Existing research on genderless fashion trends has focused on the style characteristics shown in collections and advertisements (Hong & Joo, 2020; Kim & Lee, 2016; Yang, 2020). Therefore, there is a possibility that our subjectivity was involved and consumers’ perspectives were not included. Recently, the number of consumer-led products and brands has increased remarkably; therefore, consumers’ recognition of fashion trends is critical as they affect the industry enormously. A few studies have focused on consumer reviews on fashion subjects using the big data analysis method. Lang et al. (2020) investigated consumers’ fashion rental experiences, and Choi and Lee (2020) studied ethical fashion perception. However, this is one of the first studies that deals with consumers’ preferences for genderless fashion trends by applying text-mining and LDA-based topic modeling techniques. Through this computer-aid method, researchers can extract hidden implications or estimate patterns from a natural language dataset (Hashimi et al., 2015). To analyze and understand consumers’ behaviors in real-time is becoming essential; thus, we investigated consumers’ unstructured data in fashion trends analysis.

This study has managerial implications for product planners who develop merchandise based on recent trends. We found that consumers have a high interest in brands and products related to perfume, fashion, and cosmetics in terms of genderless fashion trends that can make their individuality stand out despite gender division. Therefore, when a product planner plans a merchandising product group targeting consumers, these product categories can be prioritized. In particular, given that the topic of “perfume and scent” has been on the rise among consumers, strategic promotions and collaboration with genderless fragrance brands can also be conceived.

The limitations of this study and suggestions for future research are as follows.

This study collected the text documents from consumers postings of blogs and online communities, therefore it is not focused solely on a specific generation. Because the genderless fashion is popularly accepted by Millennials and Generation Z (Anyanwu, 2020), it would be meaningful to closely consider the opinions of various generations in the future. Continuous research is expected to be conducted in the field of fashion and textiles, because text-mining research is still scarce and at its nascency. For future research projects, it is necessary to analyze not only consumer opinions related to genderless fashion trends, but also related articles introduced in the mass media. If in-depth analyses can be conducted in the aspects of social interest and the business industry, more insights can be gained to enhance the proposed model in this study.

## Data Availability

The datasets used and analyzed during the current study are available from the first author on reasonable request.

## Abbreviations

SS: Spring summer
FW: Fall winter
